# DNA barcoding in the Southeast Pacific marine realm: Low coverage and geographic representation despite high diversity

**DOI:** 10.1371/journal.pone.0244323

**Published:** 2020-12-28

**Authors:** Jorge L. Ramirez, Ulises Rosas-Puchuri, Rosa Maria Cañedo, Joanna Alfaro-Shigueto, Patricia Ayon, Eliana Zelada-Mázmela, Raquel Siccha-Ramirez, Ximena Velez-Zuazo

**Affiliations:** 1 Facultad de Ciencias Biológicas, Universidad Nacional Mayor de San Marcos, Lima, Peru; 2 National Zoological Park, Smithsonian Conservation Biology Institute, Center for Conservation and Sustainability, Washington, DC, United States of America; 3 Department of Biological Sciences, George Washington University, Washington, DC, United States of America; 4 Facultad de Biología Marina, Universidad Científica del Sur, Lima, Perú; 5 ProDelphinus, Lima, Perú; 6 Instituto del Mar del Peru, Callao, Peru; 7 Facultad de Ciencias, Laboratorio de Genética, Fisiología y Reproducción, Universidad Nacional del Santa, Chimbote, Peru; 8 Laboratorio Costero de Tumbes, Instituto del Mar del Perú, Zorritos, Peru; 9 Asociación Peruana para la Conservación de la Naturaleza, Lima, Peru; Universidad de Magallanes, CHILE

## Abstract

The Southeast Pacific comprises two Large Marine Ecosystems, the Pacific Central-American Coastal and the Humboldt Current System; and is one of the less well known in the tropical subregions in terms of biodiversity. To address this, we compared DNA barcoding repositories with the marine biodiversity species for the Southeast Pacific. We obtained a checklist of marine species in the Southeast Pacific (i.e. Colombia, Ecuador, Chile, and Peru) from the Ocean Biodiversity Information System (OBIS) database and compared it with species available at the Barcoding of Life Data System (BOLD) repository. Of the 5504 species records retrieved from OBIS, 42% of them had at least one registered specimen in BOLD (including specimens around the world); however, only 4.5% of records corresponded to publicly available DNA barcodes including specimens collected from a Southeast Pacific country. The low representation of barcoded species does not vary much across the different taxonomic groups or within countries, but we observed an asymmetric distribution of DNA barcoding records for taxonomic groups along the coast, being more abundant for the Humboldt Current System than the Pacific Central-American Coastal. We observed high-level of barcode records with Barcode Index Number (BIN) incongruences, particularly for fishes (Actinopterygii = 30.27% and Elasmobranchii = 24.71%), reflecting taxonomic uncertainties for fishes, whereas for Invertebrates and Mammalia more than 85% of records were classified as data deficient or inadequate procedure for DNA barcoding. DNA barcoding is a powerful tool to study biodiversity, with a great potential to increase the knowledge of the Southeast Pacific marine biodiversity. Our results highlight the critical need for increasing taxonomic sampling effort, the number of trained taxonomic specialists, laboratory facilities, scientific collections, and genetic reference libraries.

## Introduction

The knowledge about the dimension of marine biodiversity remains elusive, with projected estimates of species richness varying from nearly 300 thousand [1] to over a million [2,3] or even 10 millions of species [4]. Conservative estimates for marine species that remain undescribed are between 33% to 66% [2]. This situation is no different from that of the marine biodiversity from the Southeast Pacific (SEP), one of least well-known region in the tropics [5]. This region comprises two Large Marine Ecosystems [6]: part of the Pacific Central-American Coastal encompassing the coast of Colombia, Ecuador, and the extreme north of Peru and the Humboldt Current System including the coasts of Peru and Chile. The Pacific Central-American Coastal biota is considered as highly endemic, while the Humboldt Current System has high species richness and endemism [5,7]. Globally, there is an undeniable urgency to conserve marine species and ecosystems which are under threat mostly by overexploitation and habitat loss and degradation [8], but without a better understanding of the current marine diversity, the extinction of species will pass under detected.

There are fundamental limitations for cataloguing the biodiversity, including the few taxonomy specialists working in highly diverse groups [5], limited availability of adequate facilities in coastal and research stations [5,9], and few economic incentives to conduct research on non-commercial organisms [5,9]. This all makes necessary to adopt new approaches to accelerate biodiversity inventories. In the last decade, DNA barcoding was proposed as a molecular identification tool [10] and was proved as a powerful approach to quickly asses biodiversity, including marine biota [11,12]. The use of DNA barcoding is advantageous to resolve many problems in the field of marine life studies [13], like identifying the occurrence of cryptic species which are common in marine ecosystem [11], improving identification of larvae and the relations with adult forms [13], identifying non-indigenous and potentially invasive species [14], detecting the illegal trade of regulated and protected species in processed products [15] or identifying species used in shark finning [16]. Also, DNA barcoding has a great potential for monitoring biodiversity changes (e.g. expansion/contraction of distribution ranges) caused by the disturbing influence of the periodical ENSO events [17]. The usage of DNA barcoding also offers important opportunities for species conservation and management [11] under the current biodiversity crisis scenario [14,15]. The evolution of marine biodiversity involves a series of mass extinction events, naturally [10,11], or due to anthropogenic causes [12,13]. When extinction rates of species are greater than the species discoveries, accurate species diagnosis by using DNA barcoding could support conservation efforts [18].

DNA barcoding has a great potential to assess the real dimension of the SEP marine biodiversity, a region with a high diversity of species, but with poor information available (e.g. DNA barcoding campaigns, percentage of barcoded species, low representation of taxonomic groups, or areas without data). The aim of this study was to evaluate the advance of the DNA barcoding in the SEP region, i.e. What is the coverage of barcoded species from SEP? Are there differences between taxonomic groups or geographic areas? And, what is the degree of taxonomic uncertainty revealed by the DNA barcoding data? To answer these questions, we reviewed the reports of marine species deposited in the Ocean Biogeographic Information System (OBIS) and compared them with the genetic data available in the Barcode of Life Data System (BOLD), to provide a taxonomic and geographic overview of the progress made in DNA barcoding the SEP marine biodiversity. Since many species have wide geographic distribution and the deposited DNA barcodes do not necessarily come from the SEP, we evaluated how many records are actually from specimens collected from this region. We also used the Barcode Index Number (BIN), a molecular approach to delimit Molecular Operational Taxonomic Units (MOTUs), to understand the level of hidden diversity in each evaluated taxon. It is expected that less-studied taxa will tend to have more cryptic species, and the BIN approach is an effective tool for assessing the existence of cryptic species [19]. Our study helps identify underrepresented groups, the degree of taxonomic uncertainty, and unexplored areas where more effort should be invested in future studies on biodiversity.

## Materials and methods

**Fig 1:**
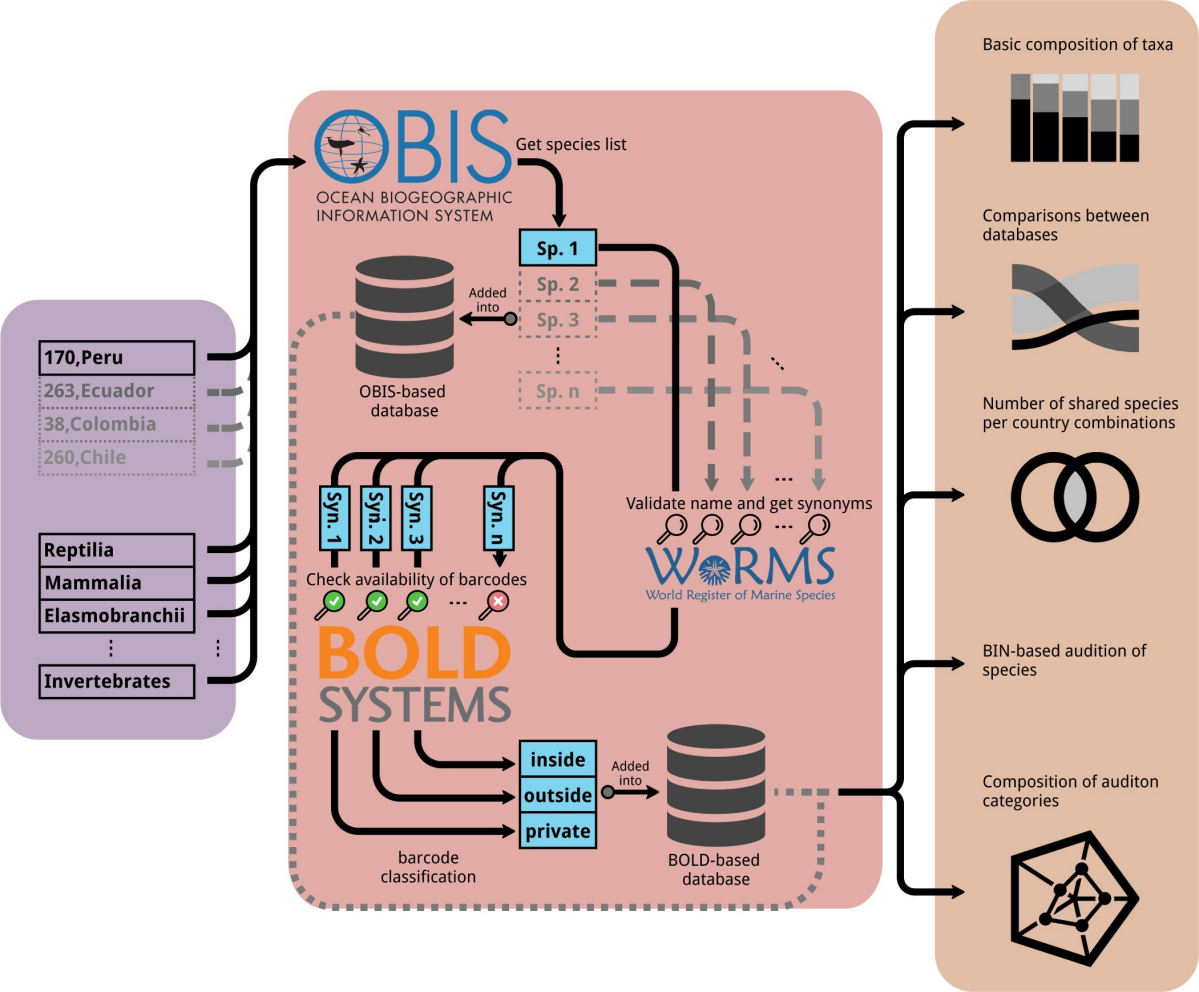
The OBc pipeline. This pipeline initializes with two files: taxa list and geographical information list, including OBIS-based area ID. Then, information from OBIS to BOLD is compared, including a step of species name validation by using WoRMS database (see main text for more details). Graphical and descriptive reports are finally generated in order to compare and contrast information generated from both OBIS and BOLD databases.

During 2019, a checklist of all marine species reported in the SEP region (i.e. Colombia, Ecuador, Peru and Chile) was obtained from OBIS database [20] and compared with the BOLD database [21] to obtain a list of species with DNA barcodes, geographic information and BIN data. For this, we developed a bioinformatic pipeline (OBc) to automatize data mining and comparisons of species between both databases (Fig 1, available at github.com/ulises-rosas/OBc). OBc starts out by downloading a list of species names by using the OBIS API (api.obis.org/v3) and two query variables: Taxon and geographic area ID (Fig 1). Species names were filtered out if a regular pattern of species nomenclature (i.e. genus + species) was not found and thus only generating species-level lists. For each name of the species lists, the currently accepted taxonomy (i.e. valid name) as well as all synonyms were obtained by using both World Register of Marine Species-WoRMS REST webservice (marinespecies.org/rest/) and web scrapping methods. A sub-list of names per valid name was created, including the valid one, and are iteratively searched for matches between record entries of BOLD by using its public API (boldsystems.org/index.php/API_Public) and then classified according to availability (i.e. either private or public) and whether it was collected in the SEP or not.

To map the geographic distribution of the records, sampling coordinates were obtained for each barcoded species available in BOLD, using BOLDmineR (github.com/ulises-rosas/boldminer). Heatmaps for distribution representing the number of records were constructed using QGis 3.4 (www.qgis.org). For multiple combinations of country-level species information, we also compared the number shared species considering three different dataset: total OBIS species, species with barcodes records, species with barcode records including at least one entry collected from the Southeast Pacific.

We performed an audition step on quality annotation (i.e. specimen metadata) for species with public DNA barcodes. The audition relies on the BOLD approach called Barcode Index Number (BIN) [22] and a classification based on Oliveira *et al*. [23], following these criteria: “A/B” when a species has more than three barcodes and their specimens are clustered on the same BIN; “C” when a species has more than three barcodes, their specimens are clustered on different BINs and those BIN only contains one species (i.e. target species); “D” when a species has less than three barcodes (i.e. data deficient); “E*” when a species has more than three barcodes, their specimens are clustered on the same BIN and that BIN contains more than one species; “E**” when a species has more than three barcodes, their specimens are clustered on different BINs and those BINs contains more than one species, and “F” when whole specimens of a species are either unvouchered or directly mined from GenBank (i.e. inadequate DNA barcoding procedure). The audition was conducted as implemented in the functions developed within OBc.

## Results

**Fig 2:**
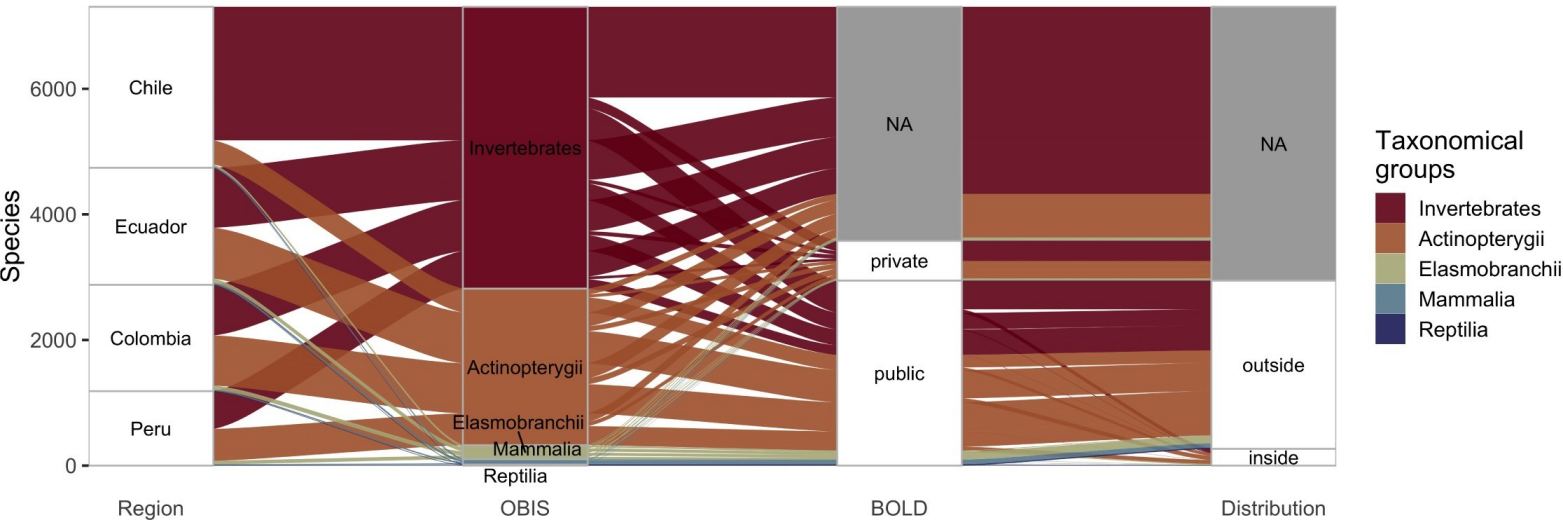
Species number decomposition through four categories: Country (i.e. region column), taxonomical group (i.e. OBIS column), availability in BOLD database (i.e. BOLD column), and distribution of barcodes relative to the Southeast Pacific (i.e. distribution column). Categories also follow the prior order. A “NA” label at columns BOLD and Distribution depicts missing data. Labels “private” and “public” at BOLD column indicate whether barcode records are private or public (at least one record), respectively. Labels “outside” and “inside” at distribution column indicate whether barcode records were obtained from specimens collected outside or inside (at least one record) the Southeast Pacific area, respectively.

We retrieved records for 5504 SEP marine species from OBIS grouped in different taxonomic groups (Table 1). The invertebrate was the group with the highest number of species reported (3890 spp), followed by bonny fishes (Actinopterygii), Elasmobranchii, Mammalia, and Reptilia. Throughout the SEP region, Chile exhibited more records, followed by Ecuador, Colombia, and Peru (Table 2, Fig 2). For Chile, the invertebrates represented 82.9% of all the marine species records for that country. In Ecuador, Colombia, and Peru, invertebrates represented ~50% of all records.

**Table 1:** Number of species from OBIS (species (OBIS)), number of barcoded species (species (BOLD)), and number of barcoded species with specimens collected from Southeast Pacific (SEP Pacific) per taxonomical group within Invertebrates and Vertebrates.

Taxonomic groups	Species (OBIS)	Species (BOLD)	Barcoded spp (%)	Barcoded SEP spp	Barcoded SEP spp (%)
**Invertebrates**					
Arthropoda	1363	403	29.6	35	2.6
Mollusca	856	314	36.7	24	2.8
Cnidaria	415	145	34.9	3	0.7
Annelida	399	109	27.3	1	0.3
Echinodermata	376	168	44.7	6	1.6
Porifera	197	24	12.2	0	0.0
Bryozoa	88	12	13.6	0	0.0
Platyhelminthes	77	0	0.0	0	0.0
Nematoda	33	1	3.0	0	0.0
Chaetognatha	22	15	68.2	0	0.0
Nemertea	21	4	19.0	2	9.5
Brachiopoda	16	1	6.3	0	0.0
Xenacoelomorpha	16	0	0.0	0	0.0
Tardigrada	4	0	0.0	0	0.0
Hemichordata	3	0	0.0	0	0.0
Acanthocephala	3	0	0.0	0	0.0
Sipuncula	1	0	0.0	0	0.0
Total	3890	1196	30.7	71	1.8
**Vertebrates**					
Actinopterygii	1439	978	68.0	155	10.8
Elasmobranchii	129	102	79.1	18	14
Mammalia	40	35	87.5	3	7.5
Reptilia	6	6	100.0	2	33.3
Total	1614	1121	69.5	178	11
**All Groups**	**5504**	**2317**	**42.1**	**249**	**4.5**

Percentages relative to OBIS species are presented. Taxonomical groups are divided in both Invertebrates and Vertebrate.

**Table 2:** Number of species from OBIS (species (OBIS)), number of barcoded species (species (BOLD)), and number of barcoded species from Southeast Pacific (SEP Pacific) per country.

Countries	Species (OBIS)	Species (BOLD)	Barcoded spp (%)	Barcoded SEP spp	Barcoded SEP spp (%)
Chile	2563	1019	39.8	119	4.6
Peru	1184	619	52.3	73	6.2
Ecuador	1863	993	53.3	80	4.3
Colombia	1695	950	56.0	2	0.1

Percentages relative to OBIS species are presented.

**Fig 3:**
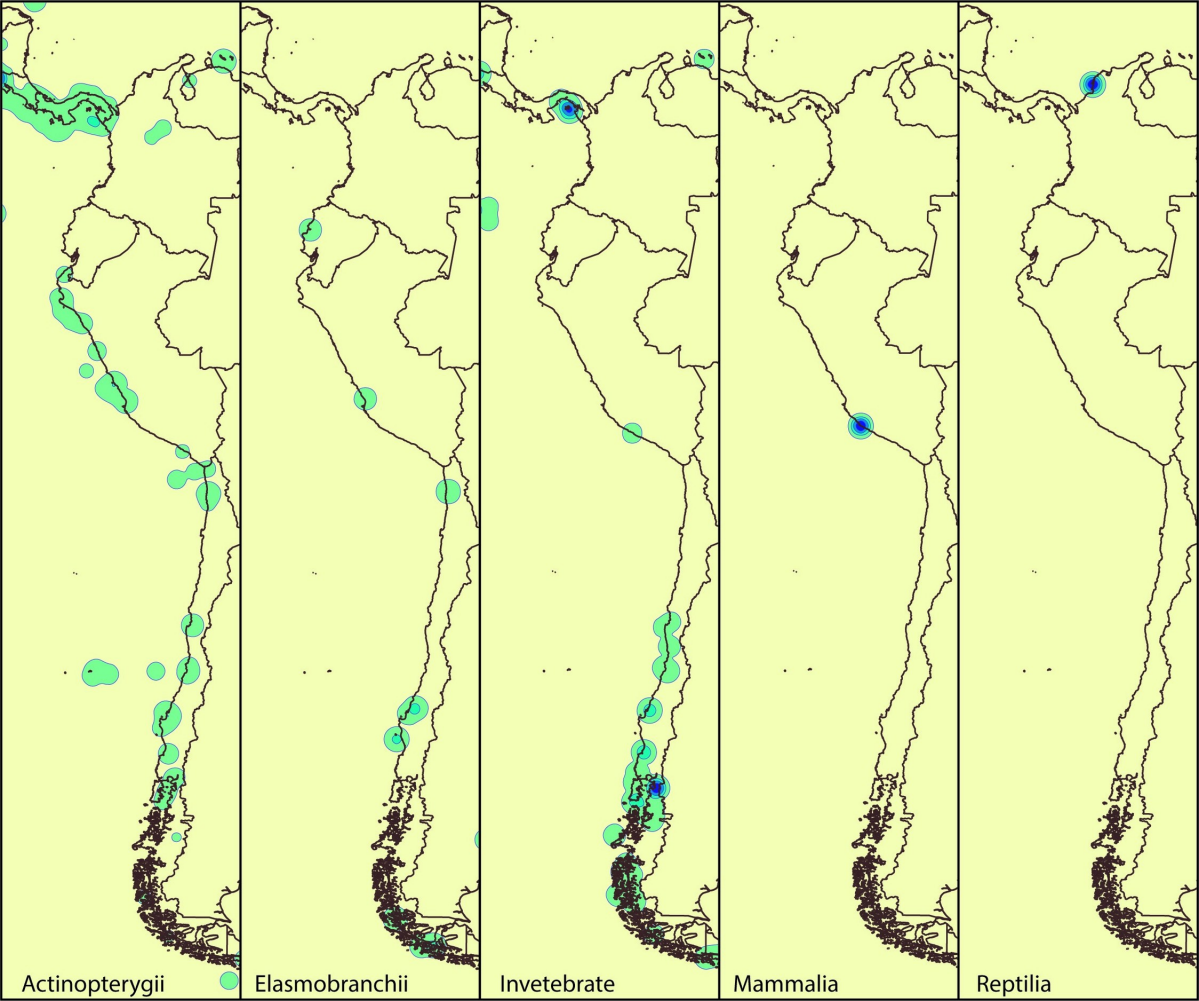
Geographical distribution of public DNA barcodes along the Southeast Pacific coast for Actinopterygii, Elasmobranchii, Invertebrate, Mammalia, and Reptilia. The circles represent localities sampling sites. Free vector data from Natural Earth (http://www.naturalearthdata.com/).

From comparing OBIS records with BOLD barcodes, we found that 42% of SEP species records found in OBIS have at least one DNA barcode deposited in BOLD; however only 4.5% of records correspond to public DNA barcodes originating in SEP countries (Fig 2). In general, Vertebrates have a higher percentage of barcoded species (68–100%) than Invertebrates (0–68.2%) (Table 1). Considering the origin of the DNA barcodes, the representation for Vertebrates and Invertebrates in the SEP decreases from 7.5–33.3% to 0–9.5%, respectively (Table 1). At a country level, the publicly DNA barcodes records were higher for Chile (119 spp), followed by Ecuador (80 spp), Peru (73 spp), and Colombia (2 spp) (Table 2). We observed more DNA barcoding records for taxonomic groups along the coasts of Chile and Peru (Humboldt Current) compared to Ecuador and Colombia (Pacific-Central American Coastal) (Fig 3).

**Fig 4:**
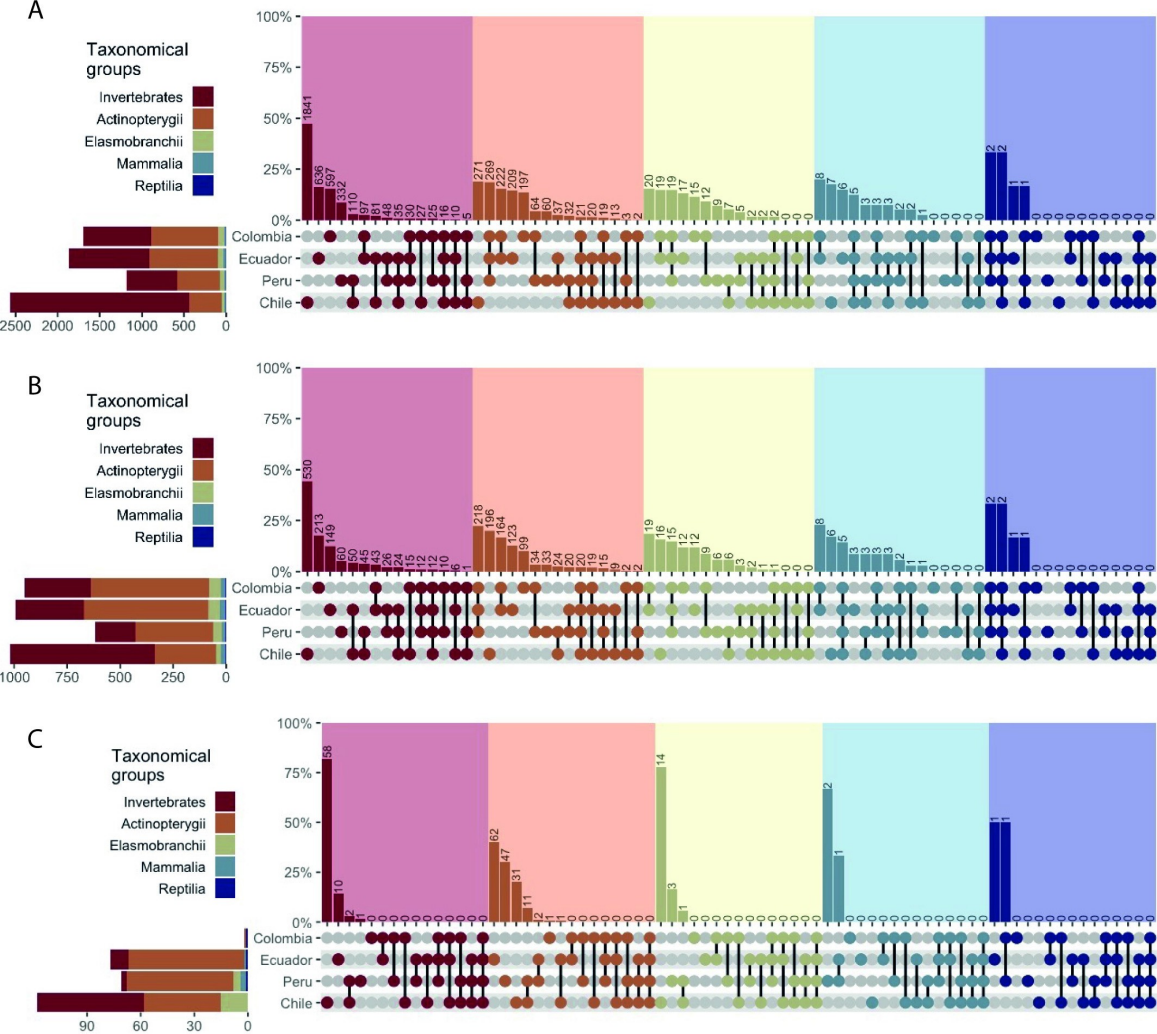
Percentage of shared species through multiple combination of countries (depicted as dumbbell plots) and per taxonomical groups for three datasets (depicted at A, B, and C). In (A) only OBIS species are considered, in (B) only barcode records are considered, and in (C) barcode records with at least one entry collected from the Southeast Pacific are considered. Numbers at the top of each bar represent the total count of shared species in a given combination. Stacked barplots at the left margin of A, B, and C represent the total number of species per country.

**Fig 5:**
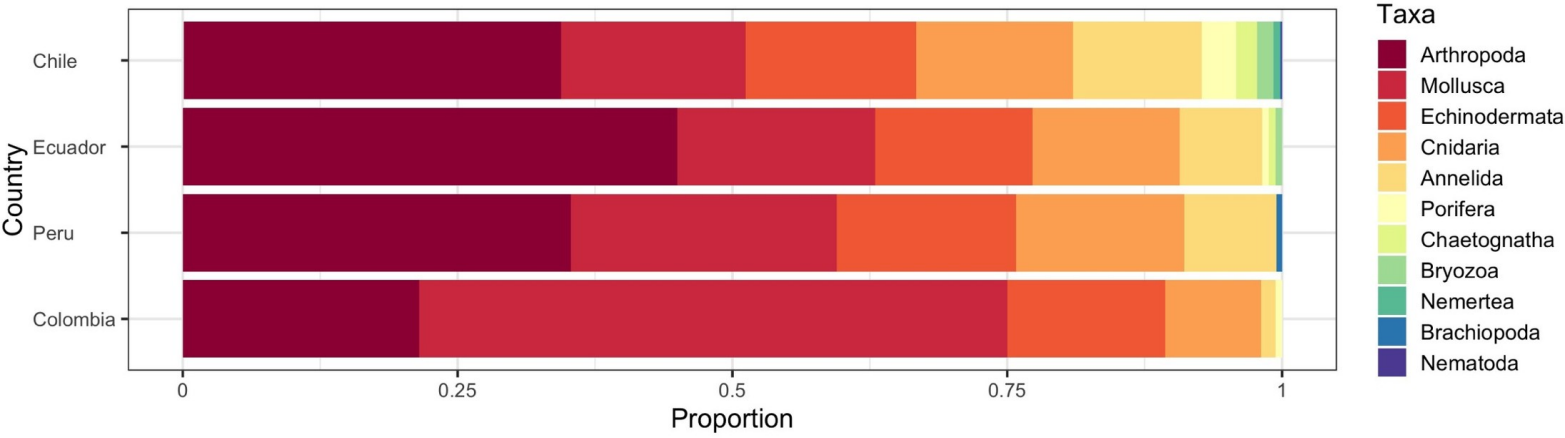
Proportions of Invertebrates taxonomical groups per country. Positions of bars are sorted in function of total number of Invertebrate species.

We estimated the number of shared OBIS species and BOLD species barcodes between countries, by taxonomic group. These values vary if: OBIS species are considered (Fig 4A), if only barcode records are considered (Fig 4B), and if only barcode records with at least one entry collected from the SEP are considered (Fig 4C). Actinopterygii is the most DNA barcoded group for Colombia (558 spp, 58.7%), Ecuador (588 spp, 59.2%) and Peru (366 spp, 59.2%), and Invertebrates for Chile (683 spp, 67%). Within Invertebrates, Arthropoda is the phylum with more species barcoded for Ecuador (145 spp, 45%), Peru (67 spp, 35.3%) and Chile (234 spp, 34.3%) while Mollusca is the group with more species barcoded for Colombia (167 spp, 53.5%) (Fig 5).

**Fig 6:**
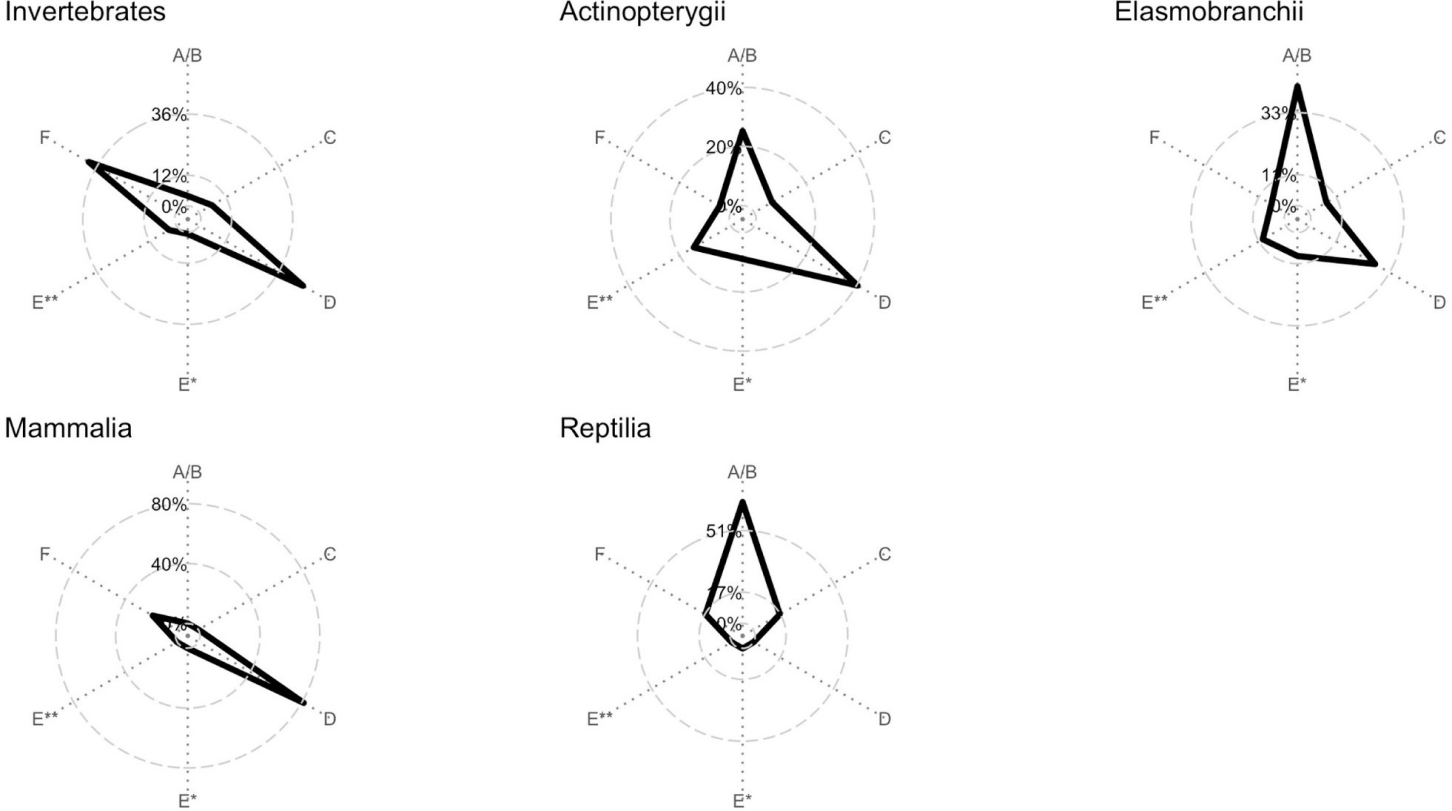
Composition of BIN-based classification (see methods for more details) for Actinopterygii, Elasmobranchii, Invertebrate, Mammalia, and Reptilia.

The audition step based on the publicly available DNA barcode data provided BIN-classification estimates for each taxon (Fig 6, S1 File). Reptilia (66.67%), Elasmobranchii (42.35%), and Actinopterygii (25.3%) were the groups with higher percentage of A/B data, species with an ideal DNA barcode. All groups, except for Reptilia, had high percentage of species classified as deficient data (D) that range from 27.06% in Elasmobranchi to 83.87% in Mammalia. Taxonomic uncertainties, the sum of C+E categories, are higher for Actinopterygii (30.27%) and Elasmobranchii (24.71%). Finally, both, Invertebrate (39.5%) and Mammalia (16.13%) exhibited barcoded species with inadequate DNA barcoding procedure (F).

## Discussion

The poor knowledge about the dimension of the marine biodiversity in certain regions of the world is the result of the combined effect of several factors including limited human and financial resources and infrastructure [5,24,25]. Cataloguing the taxonomic richness in these regions is further challenged by the increasing difficulty of detecting species that may being already experiencing displacement by climate change [26] or decimated by overexploitation and habitat loss [27,28]. In this study, we focused on the marine biodiversity in the SEP, and understudied region and, according to our results, a poorly represented region at the molecular level. Twelve years after the establishment of the International Barcode of Life Consortium (iBOL), the percentage of barcoded marine species in this region is 42%, but this estimate decreases drastically to 4.5% if only species whose barcodes originated from this region are considered. The lack of a balanced representation of barcodes across the entire geographic distribution of species will fail to capture the range of intraspecific variation, genetically structured populations or even potential cases of cryptic species [29]. The low representation of the barcoded species is consistent, and it does not vary much across the different taxonomic groups and countries (Fig 1, Tables 1 and 2). The DNA barcoding coverage could be even lower considering that the species registered in OBIS depends on specialist’s observations and the taxonomic efforts in this region are scarce [5]. The lack of specialists could further lead to several DNA barcodes records without a correct taxonomic information or an incomplete data (species only identified at genus or family level), which stresses out the need of more specialists validating the DNA barcodes being generated across the region.

Invertebrates was the group with the highest number of species records in OBIS but with the lowest barcode relative representation. While it made 61% of all species records, just 34% of them were represented by at least one DNA barcode in BOLD. This pattern was consisted across countries. Invertebrates was the group with more barcodes for all countries and this corresponded with the OBIS species distribution of species for each country. The discrepancy between number of invertebrate species reported and the barcodes is likely a result of the lack of biodiversity studies and DNA barcoding efforts, particularly in Ecuador and Peru; countries with three to eight-time less species barcoded, highlighting priority areas for future DNA barcoding research. The low DNA barcoding coverage of marine invertebrates is not exclusively for SEP and has been noted for other marine areas [29] or taxonomic groups [30]. It is worth to note that most of invertebrate species barcoded are exclusive of only one country (80% barcodes, Fig 4), highlighting the differences in invertebrate biodiversity across the region. The identification of Invertebrates can be challenging for taxonomists, delaying DNA barcoding studies [31] and limiting its potential applications on taxonomic studies [30], biomonitoring [32], and for evaluating the impacts caused by fisheries or other human activities on the ecosystems [5].

Actinopterygii is the group with more barcoded species (spp = 978), which likely reflects the commercial importance of this group [33]; however a small proportion of these records (spp = 155) had their origin in the SEP. This pattern of high coverage of fish species but low local representation was observed also in European seas, since many commercial fish have large distribution [29]. Fish species have more records georeferenced to the coasts of Peru and Chile, but not in Ecuador and Colombia (Fig 3), reflecting the magnitude of the fisheries in the southern countries [34], although it contrasts with the low within-country coverage of barcoded species estimated in 11.9% and 11.3% for Peru and Chile, respectively.

For all taxa, the distribution of DNA barcoding records is not equally represented across countries (Figs 3 and 4). For Actinopterygii, most of the barcoded species are the shared barcodes for Colombia, Ecuador, and Peru, followed by species distributed in Chile and not in the other countries (Fig 4). In all cases, SEP species reported only in Chile have high percentages of DNA barcoded species (e.g. Actinopterygii 72.3% of OBIS species), however these values vary considerably if considered only records originating from the SEP (e.g. Actinopterygii represents 16% of OBIs species), evidencing the lack of DNA barcoding campaigns in the Humboldt current. Also, the number of DNA barcoded species with records from Colombia is remarkably low (Fig 4C), indicating the urgency of efforts in this particular country. Elasmobranchii and Invertebrate were more represented in the southern part of Humboldt current than in the northern part, and scarcely in the Central-American Pacific Coastal region. In this region, the lack of barcodes for these two groups is striking, since high diversity and endemism is expected [5]. For Mammalia and Reptilia, the lack of barcodes originating in the SEP draws attention. These groups are the less diverse and the most barcoded (87.5% and 100 correspondingly), but with DNA barcodes records outside the SEP. The absence of DNA barcodes from the SEP could be due to erroneous geographic coordinates assigned to the barcodes in the database, but considering the country data registered for each BOLD record, only 33.3% of Reptilia and 7.5% of Mammalia barcodes actually originated from the SEP, evidencing the low genetic representation in this region. For Invertebrate, Actinopterygii, and Elasmobranchii, the number of reported species shared across all SEP countries is very low (Fig 4). This is expected due to the ecological and oceanographic differences between the Central American Pacific and the Humboldt Current. On the other hand, Reptilia has more species shared between countries (Fig 4) and exclusive species are uncommon, since this group was encompassed nearly exclusively by sea turtle species, which have broad distribution and are highly migratory species crossing geopolitical borders [35–37].

Several studies of DNA barcoding have found incongruences between taxonomic nominal data and MOTUs (e.g. BIN data) [23,38,39]. BIN incongruences help to highlight cases with taxonomic uncertainties, like taxonomic synonymy, cryptic diversity and specimen misidentification [22,30]. Our analysis shows that fishes, including Actinopterygii (30.27%) and Elasmobranchii (24.71%), have a higher percentage of species with BIN incongruences (C and E categories in Fig 6), reflecting the degree of taxonomic uncertainties. Previous studies have reported a high level of hidden diversity among coastal marine South American fishes [24], but mainly in Caribbean and Atlantic coasts. Here, we present a similar scenario for the SEP coast. More taxonomic effort in these groups is necessary and DNA barcoding could indicate the groups with higher taxonomic uncertainty. Reptilia also exhibited a considerable percentage of C category (16.6%); however, the number of Reptilia barcoded species (n = 6) is considerably lower than barcoded fish species (n = 200). Fishes, Invertebrates, and Mammalia have a high percentage of type F or D (Fig 6), indicating data deficient or inadequate procedure for DNA barcoding, reaching 100% in Mammalia. This can explain the fact that we observed a relatively low percentage of C+E categories for Invertebrates (9.47%). More sampling campaigns applying the DNA barcoding methodology (i.e voucher specimens and taxonomic identification by a specialist) are necessary to improve the database and achieve the benefits of this approach.

The low representation of SEP marine species with barcodes is a hurdle for DNA barcoding applications; an unknown sample cannot be identified if the species is not in the database. This has already resulted in, for example, landing species from fisheries not being correctly identified or identified at all [40] or limits its application for improved environmental baselines and monitoring [41]. Altogether, it jeopardizes efforts for identifying marine biodiversity, critical habitats and priority areas for conservation and management. For this reason, campaigns were promoted to generate DNA barcodes of taxa (e.g. FISH-BOL; http://www.fishbol.org/), ecosystems (i.e. MarBol; http://www.marinebarcoding.org/) or even countries (i.e. Peruvian Barcode of Life, PeBol; http://pebol.org/). In Peru, the consortium for DNA barcoding of Peruvian marine species (PeMar) was created in 2017 with the support of state funds (CONCYTEC) with the goal of generating a DNA barcoding reference library to study the dimension of the Peruvian marine biodiversity. PeMar is generating barcodes for 1000 species from approximately 7000 marine specimens, collected from different sources including direct sampling, directed and incidental fishery, and archived inventories. This effort will be a remarkable contribution to increase the knowledge about the diversity in the SEP since Peru is the only country that holds marine species from two subregions, the Pacific Central American and the Humboldt Current System. The study includes the validation by taxonomists of a wide variety of organisms (fish, large Vertebrates, plankton, and Invertebrates).

In conclusion, our results show the SEP as a poorly represented region at the molecular level, with a low coverage of DNA barcodes, and this does not vary much across the different taxonomic groups and countries. Also, the DNA barcode data show a high degree of taxonomic uncertainty for fishes, and data deficient or inadequate procedure for mammals and invertebrates. Despite its exploratory nature, this study offers insights into the progress of covering species with DNA barcode information in the SEP. The results of this study will help researchers across the region to focus their effort in taxonomic groups and areas less studied. More investment on research in this region are imperative, to increase sampling coverage, voucher and tissue collections, the number of trained taxonomic specialists, and appropriate laboratory facilities for taxonomic and genetic studies. Also, all data generated should be deposited on BOLD database, for open access and availability to identification tools.

## Supporting information

S1 File Data processing information. (PDF)
