# Supplementary material for: DNA barcoding in the Southeast Pacific marine realm: Low coverage and geographic representation despite high diversity
Source: PLoS One. 2020 Dec 28;15(12):e0244323. doi: 10.1371/journal.pone.0244323 (PMC7769448; doi:10.1371/journal.pone.0244323)

# DNA barcoding in the Southeast Pacific marine realm: low coverage and geographic representation despite high diversity

Jorge L. Ramirez[1*], Ulises Rosas-Puchuri[2,3], Rosa Maria Cañedo[1], Joanna Alfaro-Shigueto[4,5], Patricia Ayon[6], Eliana Zelada-Mázmela[7], Raquel Siccha-Ramirez[8], Ximena Velez-Zuazo[2,9]

[1] *Facultad de Ciencias Biológicas, Universidad Nacional Mayor de San Marcos, Lima, Peru*
[2] *Center for Conservation and Sustainability, Smithsonian Conservation Biology Institute, National Zoological Park, Washington DC, USA*
[3] *Department of Biological Sciences, George Washington University, Washington DC, USA*
[4] *Facultad de Biología Marina, Universidad Científica del Sur, Lima, Perú*
[5] *ProDelphinus, Lima, Peru*
[6] *Instituto del Mar del Peru, Callao, Peru*
[7] *Laboratorio de Genética, Fisiología y Reproducción, Facultad de Ciencias, Universidad Nacional del Santa, Chimbote, Peru*
[8] *Instituto del Mar del Perú, Laboratorio Costero de Tumbes, Zorritos, Peru*
[9] *Asociación Peruana para la Conservación de la Naturaleza, Lima, Peru*
* *e-mail: jramirezma@unmsm.edu.pe*

## S.1 Data processing

In the following, main data analyses are presented. Datasets were previously obtained with the `OBc` pipeline. Description of how `OBc` works out is shown in the main text.

### S.1.1 Setting up R requirements

We first load R libraries used throughout data processing:

```
library(dplyr) # data wrangling
library(knitr) # data frame printing
```

Upon loading libraries, main datasets are also loaded. These datasets are located here.

```
bold   = read.csv("bold.csv", stringsAsFactors = F)  # BOLD-based dataset
obis   = read.csv("obis.csv", stringsAsFactors = F)  # OBIS-based dataset
bins   = read.csv("bold_audited.tsv", sep = "\t", stringsAsFactors = F) # BIN-based dataset
```

### S.1.2 OBIS: species count per taxonomical group

Here we use the OBIS (Ocean Biogeographic Information System) dataset `obis` previously loaded. Then, we start obtaining metrics by group:

```
obis %>%
  dplyr::group_by(group, valid_name) %>%
  dplyr::summarise() %>% #getting unique values
```

```r
  dplyr::group_by(group) %>%
  dplyr::summarise(n = length(valid_name)) %>%
  dplyr::mutate(per = n*100/sum(n)) %>%
  dplyr::arrange(-n) %>%
  dplyr::ungroup() -> obis_group

knitr::kable(x = obis_group,
             digits  = 2,
             align = 'l',
             caption = "Species count
             by major taxonomical
             groups for the OBIS dataset")
```

Table 1: Species count by major taxonomical groups for the OBIS dataset

| group | n | per |
|---|---|---|
| Invertebrates | 3890 | 70.68 |
| Actinopterygii | 1439 | 26.14 |
| Elasmobranchii | 129 | 2.34 |
| Mammalia | 40 | 0.73 |
| Reptilia | 6 | 0.11 |

Obtaining metrics by country at group level:

```r
obis %>%
  dplyr::group_by(region, group, valid_name) %>%
  dplyr::summarise() %>%
  dplyr::group_by(region, group) %>%
  dplyr::summarise(n = length(valid_name)) %>%
  dplyr::mutate(per = n*100/sum(n)) %>%
  dplyr::arrange(region, -n) %>%
  dplyr::ungroup() %>%
  knitr::kable(digits  = 2,
               align = 'l',
               caption ="Species count by
               countries and major
               taxonomical groups for the OBIS dataset")
```

Table 2: Species count by countries and major taxonomical groups for the OBIS dataset

| region | group | n | per |
|---|---|---|---|
| Chile | Invertebrates | 2125 | 82.91 |
| Chile | Actinopterygii | 381 | 14.87 |
| Chile | Elasmobranchii | 33 | 1.29 |
| Chile | Mammalia | 21 | 0.82 |
| Chile | Reptilia | 3 | 0.12 |
| Colombia | Invertebrates | 807 | 47.61 |
| Colombia | Actinopterygii | 797 | 47.02 |
| Colombia | Elasmobranchii | 67 | 3.95 |
| Colombia | Mammalia | 19 | 1.12 |
| Colombia | Reptilia | 5 | 0.29 |

| region | group | n | per |
|---|---|---|---|
| Ecuador | Invertebrates | 953 | 51.15 |
| Ecuador | Actinopterygii | 810 | 43.48 |
| Ecuador | Elasmobranchii | 66 | 3.54 |
| Ecuador | Mammalia | 29 | 1.56 |
| Ecuador | Reptilia | 5 | 0.27 |
| Peru | Invertebrates | 601 | 50.76 |
| Peru | Actinopterygii | 506 | 42.74 |
| Peru | Elasmobranchii | 56 | 4.73 |
| Peru | Mammalia | 16 | 1.35 |
| Peru | Reptilia | 5 | 0.42 |

### S.1.3 BOLD: basic metrics

Here we use the BOLD (Barcode of Life System) dataset `bold` previously loaded. Then, we start obtaining metrics by group:

```
obis_totalspps = length(unique(obis$valid_name))
bold_totalspps = length(unique(bold$valid_name))
```

Percentage of species having a record in BOLD:

```
bold_totalspps*100/obis_totalspps
```

```
## [1] 42.09666
```

Percentage of BOLD species that have public record inside the study area:

```
length(
  unique(
    bold["public_inside" == bold$availability,"valid_name"]
    )
  )*100/obis_totalspps
```

```
## [1] 4.523983
```

Percentage of BOLD species that have public record outside the study area:

```
length(
  unique(
    bold["public_outside" == bold$availability,"valid_name"]
    )
  )*100/obis_totalspps
```

```
## [1] 32.2311
```

Obtaining metrics by group

```
bold %>%
  dplyr::group_by(group, valid_name) %>%
  dplyr::summarise() %>% #getting unique values
  dplyr::group_by(group) %>%
  dplyr::summarise(n = length(valid_name)) %>%
  dplyr::mutate(per = n*100/sum(n)) %>%
  dplyr::arrange(-n) %>%
  dplyr::ungroup() -> bold_group

knitr::kable(x = bold_group,
```

```
              digits  = 2,
              align = 'l',
              caption = "Species count
              by major taxonomical
              groups for the BOLD dataset")
```

Table 3: Species count by major taxonomical groups for the BOLD dataset

| group | n | per |
|---|---|---|
| Invertebrates | 1196 | 51.62 |
| Actinopterygii | 978 | 42.21 |
| Elasmobranchii | 102 | 4.40 |
| Mammalia | 35 | 1.51 |
| Reptilia | 6 | 0.26 |

Obtaining metrics by country at group level:

```
bold %>%
  dplyr::group_by(region, group, valid_name) %>%
  dplyr::summarise() %>%
  dplyr::group_by(region, group) %>%
  dplyr::summarise(n = length(valid_name)) %>%
  dplyr::mutate(per = n*100/sum(n)) %>%
  dplyr::arrange(region, -n) %>%
  dplyr::ungroup() %>%
  knitr::kable(digits  = 2,
               align = 'l',
               caption ="Species count by
               countries and major
               taxonomical groups for the BOLD dataset" )
```

Table 4: Species count by countries and major taxonomical groups for the BOLD dataset

| region | group | n | per |
|---|---|---|---|
| Chile | Invertebrates | 683 | 67.03 |
| Chile | Actinopterygii | 287 | 28.16 |
| Chile | Elasmobranchii | 26 | 2.55 |
| Chile | Mammalia | 20 | 1.96 |
| Chile | Reptilia | 3 | 0.29 |
| Colombia | Actinopterygii | 558 | 58.74 |
| Colombia | Invertebrates | 312 | 32.84 |
| Colombia | Elasmobranchii | 56 | 5.89 |
| Colombia | Mammalia | 19 | 2.00 |
| Colombia | Reptilia | 5 | 0.53 |
| Ecuador | Actinopterygii | 588 | 59.21 |
| Ecuador | Invertebrates | 322 | 32.43 |
| Ecuador | Elasmobranchii | 53 | 5.34 |
| Ecuador | Mammalia | 25 | 2.52 |
| Ecuador | Reptilia | 5 | 0.50 |
| Peru | Actinopterygii | 366 | 59.22 |
| Peru | Invertebrates | 190 | 30.74 |

| region | group | n | per |
|---|---|---|---|
| Peru | Elasmobranchii | 42 | 6.80 |
| Peru | Mammalia | 15 | 2.43 |
| Peru | Reptilia | 5 | 0.81 |

Obtaining metrics by country at subgroup level for Invertebrates

```
bold %>%
  dplyr::filter(grepl("Invertebrates", group)) %>%
  dplyr::group_by(region, subgroup, valid_name) %>%
  dplyr::summarise() %>%
  dplyr::group_by(region, subgroup) %>%
  dplyr::summarise(n = length(valid_name)) %>%
  dplyr::mutate(per = n*100/sum(n)) %>%
  dplyr::arrange(region, -n) %>%
  dplyr::ungroup() %>%
  knitr::kable(digits  = 2,
             align = 'l',
             caption ="Species count
             by countries and Invertebrates
             subgroups for the BOLD dataset" )
```

Table 5: Species count by countries and Invertebrates subgroups for the BOLD dataset

| region | subgroup | n | per |
|---|---|---|---|
| Chile | Arthropoda | 234 | 34.26 |
| Chile | Mollusca | 115 | 16.84 |
| Chile | Echinodermata | 106 | 15.52 |
| Chile | Cnidaria | 98 | 14.35 |
| Chile | Annelida | 80 | 11.71 |
| Chile | Porifera | 21 | 3.07 |
| Chile | Chaetognatha | 13 | 1.90 |
| Chile | Bryozoa | 10 | 1.46 |
| Chile | Nemertea | 4 | 0.59 |
| Chile | Brachiopoda | 1 | 0.15 |
| Chile | Nematoda | 1 | 0.15 |
| Colombia | Mollusca | 167 | 53.53 |
| Colombia | Arthropoda | 67 | 21.47 |
| Colombia | Echinodermata | 45 | 14.42 |
| Colombia | Cnidaria | 27 | 8.65 |
| Colombia | Annelida | 4 | 1.28 |
| Colombia | Porifera | 2 | 0.64 |
| Ecuador | Arthropoda | 145 | 45.03 |
| Ecuador | Mollusca | 58 | 18.01 |
| Ecuador | Echinodermata | 46 | 14.29 |
| Ecuador | Cnidaria | 43 | 13.35 |
| Ecuador | Annelida | 24 | 7.45 |
| Ecuador | Bryozoa | 2 | 0.62 |
| Ecuador | Chaetognatha | 2 | 0.62 |
| Ecuador | Porifera | 2 | 0.62 |
| Peru | Arthropoda | 67 | 35.26 |
| Peru | Mollusca | 46 | 24.21 |

| region | subgroup | n | per |
|---|---|---|---|
| Peru | Echinodermata | 31 | 16.32 |
| Peru | Cnidaria | 29 | 15.26 |
| Peru | Annelida | 16 | 8.42 |
| Peru | Brachiopoda | 1 | 0.53 |

### S.1.4 BIN: basic metrics

Here we use the BIN (Barcode Index Number) dataset `bins` previously loaded. Then, we start obtaining metrics of BIN-based classification per taxonomical groups:

```
bins %>%
  dplyr::group_by(Group, Classification, Species) %>%
  dplyr::summarise() %>%
  dplyr::group_by(Group, Classification) %>%
  dplyr::summarise(n = length(Classification)) %>%
  dplyr::mutate(per = n*100/sum(n)) %>%
  dplyr::ungroup() %>%
  knitr::kable(digits  = 2,
               align = 'l',
               caption ="Composition of BIN-based
               classification by
               major taxonomical groups")
```

Table 6: Composition of BIN-based classification by major taxonomical groups

| Group | Classification | n | per |
|---|---|---|---|
| Actinopterygii | A | 148 | 17.92 |
| Actinopterygii | B | 61 | 7.38 |
| Actinopterygii | C | 57 | 6.90 |
| Actinopterygii | D | 331 | 40.07 |
| Actinopterygii | E* | 73 | 8.84 |
| Actinopterygii | E** | 120 | 14.53 |
| Actinopterygii | F | 36 | 4.36 |
| Elasmobranchii | A | 31 | 36.47 |
| Elasmobranchii | B | 5 | 5.88 |
| Elasmobranchii | C | 6 | 7.06 |
| Elasmobranchii | D | 23 | 27.06 |
| Elasmobranchii | E* | 7 | 8.24 |
| Elasmobranchii | E** | 8 | 9.41 |
| Elasmobranchii | F | 5 | 5.88 |
| Invertebrates | A | 12 | 1.31 |
| Invertebrates | B | 24 | 2.61 |
| Invertebrates | C | 53 | 5.77 |
| Invertebrates | D | 433 | 47.12 |
| Invertebrates | E* | 5 | 0.54 |
| Invertebrates | E** | 29 | 3.16 |
| Invertebrates | F | 363 | 39.50 |
| Mammalia | D | 26 | 83.87 |
| Mammalia | F | 5 | 16.13 |
| Reptilia | A | 4 | 66.67 |
| Reptilia | C | 1 | 16.67 |

| Group | Classification | n | per |
|---|---|---|---|
| Reptilia | F | 1 | 16.67 |

Collapsing Actinopterygii and Elasmobranchii as fishes:

```
bins$Group <- gsub("(Actinopterygii|Elasmobranchii)", "Fishes", bins$Group)

bins %>%
  dplyr::group_by(Group, Classification, Species) %>%
  dplyr::summarise() %>%
  dplyr::group_by(Group, Classification) %>%
  dplyr::summarise(n = length(Classification)) %>%
  dplyr::mutate(per = n*100/sum(n)) %>%
  dplyr::ungroup() %>%
  knitr::kable(digits  = 2,
              align = 'l',
              caption ="Same as Table 6 expect that
              both Actinopterygii and Elasmobranchii
              are collapsed within the Fishes group")
```

Table 7: Same as Table 6 expect that both Actinopterygii and Elasmobranchii are collapsed within the Fishes group

| Group | Classification | n | per |
|---|---|---|---|
| Fishes | A | 179 | 19.65 |
| Fishes | B | 66 | 7.24 |
| Fishes | C | 63 | 6.92 |
| Fishes | D | 354 | 38.86 |
| Fishes | E* | 80 | 8.78 |
| Fishes | E** | 128 | 14.05 |
| Fishes | F | 41 | 4.50 |
| Invertebrates | A | 12 | 1.31 |
| Invertebrates | B | 24 | 2.61 |
| Invertebrates | C | 53 | 5.77 |
| Invertebrates | D | 433 | 47.12 |
| Invertebrates | E* | 5 | 0.54 |
| Invertebrates | E** | 29 | 3.16 |
| Invertebrates | F | 363 | 39.50 |
| Mammalia | D | 26 | 83.87 |
| Mammalia | F | 5 | 16.13 |
| Reptilia | A | 4 | 66.67 |
| Reptilia | C | 1 | 16.67 |
| Reptilia | F | 1 | 16.67 |

### S.1.5 OBIS and BOLD: comparative table

Rough comparison

```
colnames(bold_group)[2:3] <- c("n1", "per1")

dplyr::left_join(obis_group, bold_group, by = "group") %>%
  dplyr::select(group, n, n1) %>%
```

```
dplyr::mutate(per = n1*100/n) %>%
dplyr::arrange(-per) %>%
knitr::kable(digits  = 2,
             align = 'l',
             caption ="Comparison between both
             OBIS and BOLD datasets")
```

Table 8: Comparison between both OBIS and BOLD datasets

| group | n | n1 | per |
|---|---|---|---|
| Reptilia | 6 | 6 | 100.00 |
| Mammalia | 40 | 35 | 87.50 |
| Elasmobranchii | 129 | 102 | 79.07 |
| Actinopterygii | 1439 | 978 | 67.96 |
| Invertebrates | 3890 | 1196 | 30.75 |

## S.2 Data visualization at Unix terminal

Make sure `OBc` is isntalled.

### S.2.1 Upset plot

Stripes (`-l`) and blocks (`-b`) options are explicitly stated to keep consistancy in each plot

For entire dataset of bold

```
upsetplot -i data/bold.csv\
          -l "Colombia,Ecuador,Peru,Chile"\
          -b "Invertebrates,Actinopterygii,Elasmobranchii,Mammalia,Reptilia"
```

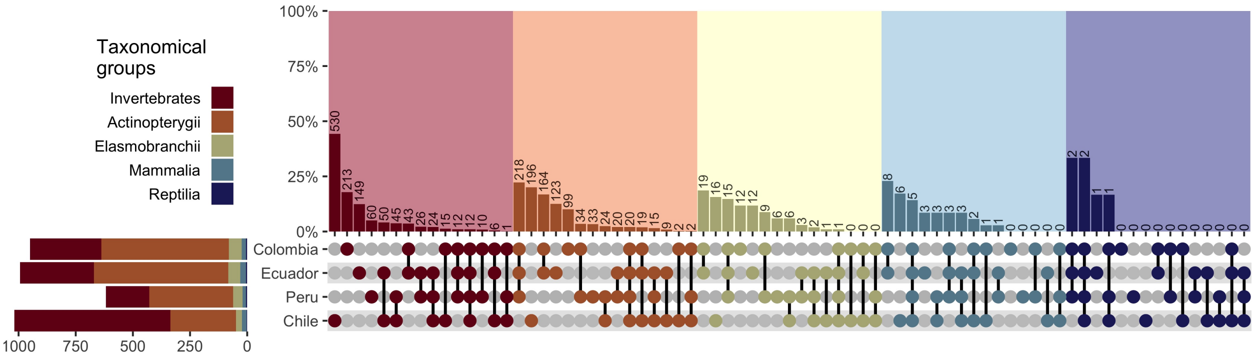


For entire dataset of obis

```
upsetplot -i data/obis.csv\
          -l "Colombia,Ecuador,Peru,Chile"\
          -b "Invertebrates,Actinopterygii,Elasmobranchii,Mammalia,Reptilia"
```

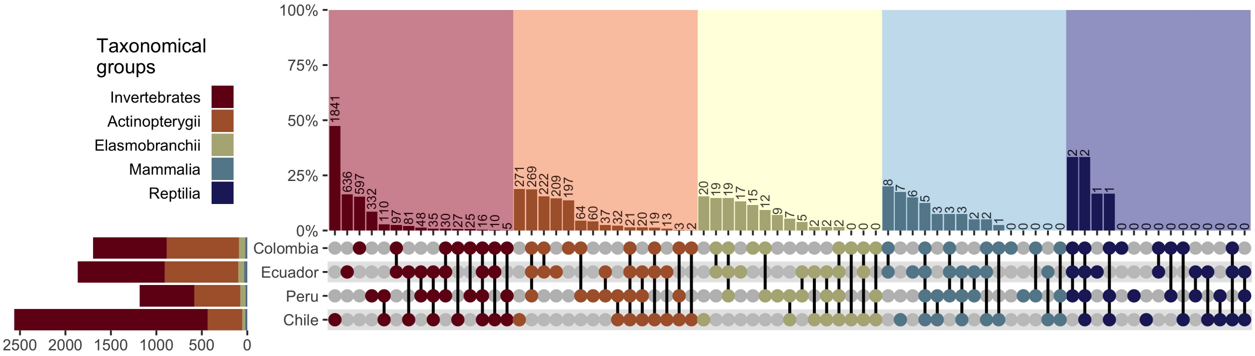


Only for species with public BOLD record and distributed inside the study area

```
# filter records by availability
cat data/bold.csv | grep "public_inside" > data/bold_public_inside.csv

upsetplot -i data/bold_public_inside.csv\
          -l "Colombia,Ecuador,Peru,Chile"\
          -b "Invertebrates,Actinopterygii,Elasmobranchii,Mammalia,Reptilia"
```

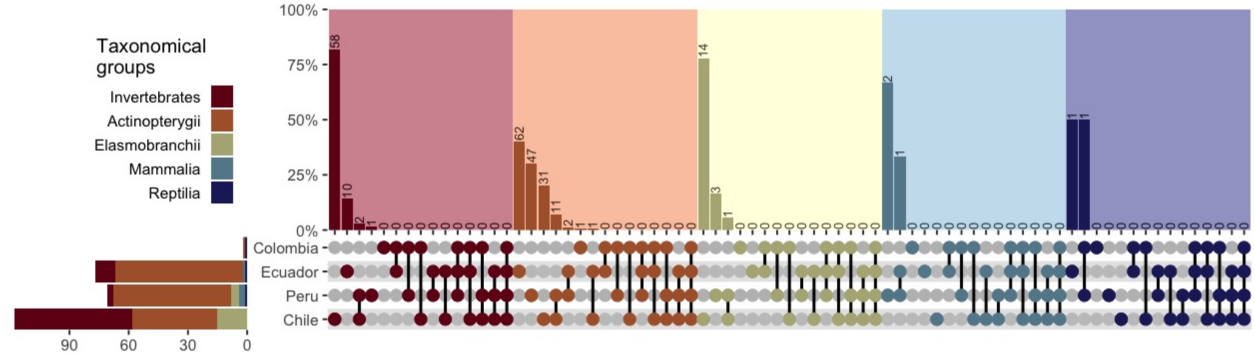

Supplement: S1 File — (PDF) [file pone.0244323.s001.pdf]
